# Regulation of CD4 T Cell Responses by the Transcription Factor Eomesodermin

**DOI:** 10.3390/biom12111549

**Published:** 2022-10-24

**Authors:** Kunal Dhume, Brandon Kaye, K. Kai McKinstry

**Affiliations:** Immunity and Pathogenesis Division, Burnett School of Biomedical Sciences, College of Medicine, University of Central Florida, Orlando, FL 34744, USA

**Keywords:** CD4 T cell, Eomesodermin, Th subsets, cytolytic CD4 T cell, Th1, Th17

## Abstract

Central to the impacts of CD4 T cells, both positive in settings of infectious disease and cancer and negative in the settings of autoimmunity and allergy, is their ability to differentiate into distinct effector subsets with specialized functions. The programming required to support such responses is largely dictated by lineage-specifying transcription factors, often called ‘master regulators’. However, it is increasingly clear that many aspects of CD4 T cell immunobiology that can determine the outcomes of disease states involve a broader transcriptional network. Eomesodermin (Eomes) is emerging as an important member of this class of transcription factors. While best studied in CD8 T cells and NK cells, an increasing body of work has focused on impacts of Eomes expression in CD4 T cell responses in an array of different settings. Here, we focus on the varied impacts reported in these studies that, together, indicate the potential of targeting Eomes expression in CD4 T cells as a strategy to improve a variety of clinical outcomes.

## 1. Introduction

While once thought to be largely restricted to providing ‘help’ to B cells and sometimes CD8 T cells, CD4 T cells are now understood to impact immune responses in a variety of different ways. These modes of action include acting as cytolytic effector cells, as regulators of immune responses, and as orchestrators of innate inflammation, in addition to their more traditional helper activities [1]. CD4 T cells must adopt specialized activation states that are largely directed by the actions of different transcription factors to fulfill these diverse roles. The most important of these transcription factors are so-called ‘master regulators’ that are required for CD4 T cells activated by antigens to express a set of characteristics that define archetypal ‘Th’ subsets. The best-defined Th subsets include Th1, Th2, Th17, and Tregs, which require expression of the ‘master regulators’ T-bet, Gata3, Rorγt, and FoxP3, respectively. The generation of these different subsets is critical for effective immune responses against specific kinds of threats. For example, Th1 cells coordinate efficient clearance of intracellular pathogens and cancers, Th2 cells enable the elimination of extracellular pathogens, and Th17 cells are a prominent component in the defense against fungal pathogens. When turned against self or environmental antigens, these same subsets can promote autoimmune and allergic conditions.

It is increasingly clear that transcription factors beyond these ‘master regulators’ play important roles in CD4 T cell activation, differentiation, function, and memory fate [2,3]. Therefore, many of these transcription factors hold promise as targets to tailor specific aspects of CD4 T cell functionality and are thus receiving increasing attention in animal and clinical studies. Here, we will focus our discussion on how one such transcription factor, Eomesodermin (Eomes), has been found to impact CD4 T cell responses in a variety of different settings. Eomes is a member of the T-box family of transcription factors, along with T-bet (the Th1 ‘master regulator’). Unlike T-bet’s restricted role in haemopoietic cells, Eomes has an important role in cells outside of the immune system. Notably, Eomes deficiency results in embryonic lethality [4], resulting in an obstacle for earlier studies utilizing traditional ‘knockout’ mouse models to determine its role in immune cells. However, seminal studies using ectopic expression of a dominant negative version of T-bet in CD8 T cells revealed the presence of another T-bet-like transcription factor able to regulate hallmark attributes associated with effective CD8 T cell responses, which was confirmed to be Eomes [5]. Subsequent studies employing Eomes overexpression and more recently developed conditional Eomes knockout mouse models have revealed important roles for Eomes in CD8 T cells, natural killer (NK) cells, and innate lymphoid cells [5,6,7].

This discussion will be limited to studies addressing the role of Eomes in CD4 T cells, as a complex body of literature has developed in recent years. Specifically, we will focus on (i) the regulation of Eomes expression in activated CD4 T cells, (ii) the functional attributes of CD4 T cells that can be impacted by Eomes, and (iii) the diverse array of disease states reported to be impacted by Eomes expression in CD4 T cells. Together, these studies reveal varied actions of Eomes that span across different Th subsets, which can dramatically impact outcomes in animal models and in the clinic. Additionally, we speculate on roles for Eomes in modulating memory fate and CD4 T cell exhaustion based partially on evidence for Eomes as regulators of these complex processes in CD8 T cells. Elucidating further mechanisms regulating Eomes in CD4 T cells, and how its actions intersect with other transcription factors to impact function and survival fitness may offer powerful new avenues to improve vaccination and immunotherapy. 

## 2. Regulation of Eomes Expression during CD4 T Cell Activation

**Figure 1:**
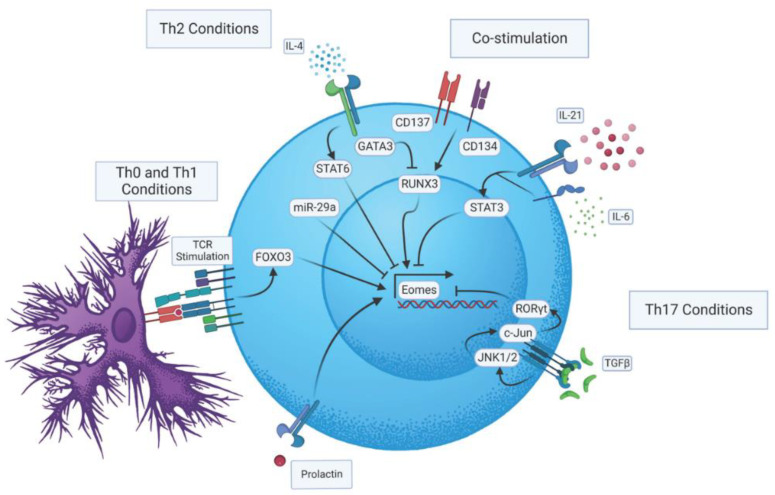
Regulatory pathways impacting Eomes expression in CD4 T cells.

Like most transcription factors impacting CD4 T cell responses, Eomes is regulated by multiple mechanisms that we will discuss in the following sections (these are summarized in Figure 1). Furthermore, differences in Eomes levels between naive, effector, and memory states in CD4 T cells indicate that distinct mechanisms may impact its expression depending on a cell’s activation state [8]. Antigen stimulation via the T cell receptor (TcR) (signal one) during T cell activation is a critical regulator of Eomes induction, as it is nearly undetectable in naïve CD4 T cells [5,9,10]. In fact, Eomes expression upon TcR stimulation has been shown to be dose dependent [9]. CD4 T cells deficient in the forkhead transcription factor, forkhead box O-3 (Foxo3), itself positively regulated by TcR stimulation, are marked by reduced Eomes expression [11]. The importance of Eomes regulation by Foxo3 will be discussed in more detail further on. Interleukin-2 (IL-2), the critical autocrine T cell growth factor induced by TcR stimulation, does not seem to directly regulate Eomes, as CD25- and IL-2-deficient CD4 T cells can upregulate Eomes upon antigen stimulation [9,12]. Interestingly, though, CD4 T cells deficient in Signal Transducer and Activator of Transcription 5 (STAT5), an important mediator of IL-2 signaling, are marked by increased Eomes expression [13]. This may indicate that other signaling molecules that function via STAT5 may repress Eomes in CD4 T cells during their activation in at least some inflammatory settings. Co-stimulatory molecules (signal two), notably 4-1BB (CD137) and OX-40 (CD134), belonging to the Tumor Necrosis Factor (TNF) receptor superfamily, can boost Eomes expression in activated CD4 T cells during priming. Moreover, dual CD137 and CD134 co-stimulation can enhance Eomes several fold with no concomitant impact on T-bet [12,14,15,16,17]. Thus, the strength of the antigen signal and the array of costimulatory interactions engaged during activation appear to be critical in fine-tuning Eomes induction during the initial phases of CD4 T cell activation.

**Figure 2:**
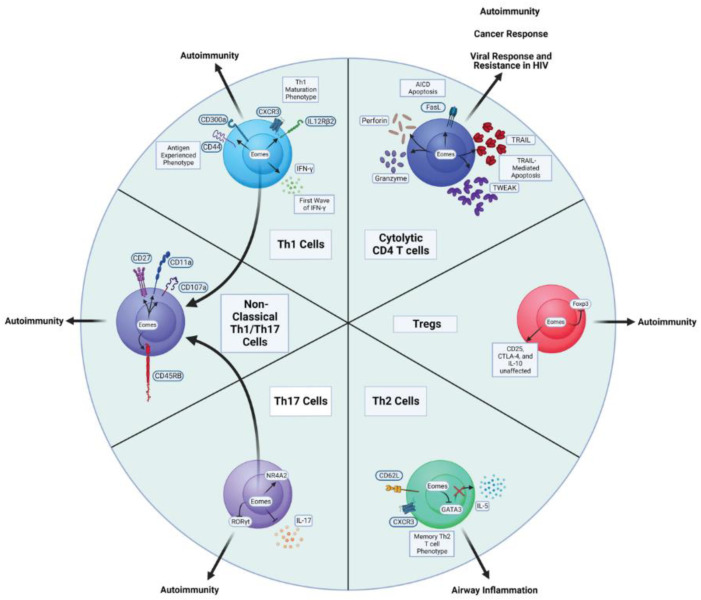
Major CD4 T cell subsets impacted by Eomes expression and disease states associated with them.

Despite being most strongly associated with Th1 and cytolytic CD4 T cell attributes, Eomes can impact the functional capacity of several other Th subsets, as described below and highlighted in Figure 2. The varied impacts that have been attributed to Eomes may reflect different integrations of the multivariate modes of Eomes regulation that have been defined partly through studies employing well-defined CD4 T cell subsets (Figure 1). It is also likely that the aspects of Eomes-dependent regulation highlighted below reflect integration with other programs (some specific to individual Th subsets) operating in CD4 T cell effectors. In the following sections, we will summarize findings indicating how Eomes is regulated in major effector CD4 T cell subsets, and how its induction has been reported to impact their function.

## 3. Eomes and Th1 Cells

Under well-controlled conditions in vitro, maximal Eomes expression is seen in Th1 versus Th2, Th17, or Th0 polarizing conditions [18]. It is still unclear, though, to what extent individual Th1 regulators affect Eomes expression, as contradictory results have been reported. For example, Eomes induction was shown to be independent of STAT1 or STAT4 activation [9], the transcriptional enforcers of interferon gamma (IFNγ) and IL-12 signaling, respectively, that are required for maximal T-bet induction and full Th1-polarization [19,20]. However, in other studies, reduced Eomes expression was seen in CD4 T cells deficient in either STAT1 or STAT4 when cultured in neutral conditions [9]. The reasons for these discrepancies are not entirely clear, but may relate to differences in the culture conditions employed. In another study, CD4 T cells stimulated with and without IL-12 (that activates STAT4) had no differences in Eomes expression [21], but Th17 clones grown in presence of IL-12 showed increased Eomes. This suggests that Th-differentiation states may dictate how individual cytokines impact Eomes expression in activated CD4 T cells. It is noteworthy that IL-12 seems to have a strong negative impact on Eomes expression CD8 T cells [22], suggesting that control of Eomes may also differ in important ways between CD4 and CD8 T cell responses. 

Runt-related transcription factor 3 (Runx3), another regulator linked to Th1 polarization [23], appears to be a strong inducer of Eomes. This is clearly seen in CD4 T cells ectopically expressing Runx3 that show minimal T-bet expression but substantial Eomes induction [24]. Additionally, dual CD137/134 co-stimulation leading to increased Eomes appears to be Runx3-dependent [12]. Interestingly though, cells grown under Th2 conditions, but deficient for Gata-3, have been shown to express more Eomes than when cultured in Th1 conditions [24]. This suggests that Eomes is repressed by certain Th1-specific regulatory elements. One such mechanism may involve regulation by microRNA (miRNA). For example, miR-29 deficiency in CD4 T cells resulted in a ~70% increase of Eomes expression, whereas miR-29 introduction resulted in a ~80% reduction in expression [25]. More recently, miR-92a has also been shown to negatively regulate Eomes in CD4 T cells using similar techniques [26]. 

Eomes has been found, in many situations, to have a positive impact on Th1 functional attributes. Eomes can directly regulate IFNγ production in CD4 T cells by transactivating the *IFNG* promoter [11]. However, unlike other regulators of Th1 polarization, Eomes can also induce IFNγ production in TcR-stimulated CD4 T cells under non-polarizing conditions [11]. In this regard, Eomes can precede, and thus, indirectly upregulate T-bet expression by promoting an initial wave of IFNγ production after TcR stimulation [11,27]. Furthermore, T cell-specific deletion of Eomes was found to reduce IFNγ production by CD4 T cells, which could be restored by ectopic expression of Eomes [11]. In line with a role for Eomes in maximizing IFNγ production, human Th1 cells marked by expression of CD300a and high Eomes were found to produce more IFNγ and more TNF and IL-2 (also Th1-associated cytokines) than Th1 cells not able to upregulate Eomes after stimulation [28]. It is important to note, though, that ectopic expression of Eomes does not result in IFNγ production as robust as is seen cells expressing high T-bet [21,25].

Besides IFNγ, Eomes can positively regulate other key factors associated with Th1 responses. Optimal Th1 development requires the expression of the IL-12 receptor for STAT4-mediated Th1 imprinting of cells [27]. Additionally, high expression of the chemokine receptor CXCR3 is associated with optimal Th1 CD4 trafficking to sites of inflammation [29]. Yagi et al. reported that Eomes expression promoted the expression of both IL-12Rβ2 and CXCR3 in the absence of T-bet or IL-12 in Gata-3-deficient CD4 T cells grown under Th2 conditions [24]. Thus, while T-bet and IL-12-mediated STAT4 signaling are critical for enforcing Th1 development in CD4 T cells, Eomes can compensate, to some extent, in their absence.

## 4. Eomes and Cytolytic CD4 T Cells

CD4 T cell effectors are found to mediate direct cytolytic activity in an increasing number of settings [30,31,32,33,34]. Key effector mechanisms responsible for their cytolytic activity mirror the major pathways underlying CD8 T cell cytotoxicity. First, cytolytic CD4 T cells often express Fas ligand (FasL), which, on binding with the Fas-receptor on target cells, leads to activation of a caspase-mediated apoptosis pathway [35,36]. Second, CD4 T cells can mediate cytolytic activities through the release of toxic granules containing perforin and granzymes, triggering target cell death [37,38,39,40]. Third, CD4 T cells can mediate killing through TNF-related apoptosis-inducing ligand (TRAIL)-mediated pathways [41,42]. While the transcriptional control of cytolytic activity in CD4 T cells is still only partially understood, several studies suggest that Eomes may play a central role in directing this Th lineage [43,44]. 

Seminal studies found that overexpression of Eomes in Th2 effectors led to increases in both perforin and Granzyme B mRNA expression [5]. In addition, ectopic Eomes expression was found to render transfected cells susceptible to death even in the presence of Concanamycin A, an inhibitor of lysosomal acidification required for cytotoxic granule release [45], indicating that the cell death was mediated by mechanisms independent of perforin and Granzyme B [44]. Indeed, the transfected Eomes^+^ cells were shown to express FasL, and their ability to kill was completely abrogated by the addition of FasL blocking antibodies. Moreover, the killing efficiency of Eomes-transfected cells was higher than that of controls with ectopic perforin expression, indicating that Eomes has broader roles in mediating cytotoxicity [44]. 

Several studies implicate CD134 and CD137 co-stimulation, which, as discussed above, promotes Eomes expression as critical for the development of cytotoxic potential in CD4 T cells [12,14,15,16,17]. Interestingly, the relative expression level of T-bet vs Eomes may help identify effector cells with the greatest cytolytic activity in mixed populations. For example, Choi et al. identified three distinct effector populations based on the expression of Eomes and T-bet: Eomes^+^T-bet^-^ expressed robust cytotoxic activity, while T-bet^+^Eomes^-^ or T-bet^+^Eomes^+^ were exclusively Th1, or were intermediate between a Th1 and cytotoxic phenotype, respectively [17].

It is important to note that regulation of CD4 T cell cytotoxic capacity has also been shown to be controlled by differential expression of transcription factors other than Eomes including T cell Factor 1 (Tcf-1) and B lymphocyte-induced maturation protein-1 (Blimp-1) [46], as well as Runx3 and T helper-inducing POZ/Krueppel-like factor (ThPOK) [47,48]. This suggests that distinct subsets of cytotoxic CD4 T cells may be promoted by different sets of inflammatory signals, and that these may be marked by unique phenotypes and/or functional capacity. For example, Gruarin et al. identified a subset of human CD4 T cells with both regulatory and cytolytic potential expressing higher Eomes and lower T-bet expression than conventional cytolytic effectors. Most of the former cells were marked by strong Granzyme K expression, with ~30% also expressing Granzyme B, while virtually all cells in the conventional cytotoxic subset were Granzyme B+ with only ~10% expressing Granzyme K^+^ [49]. Such heterogeneity implies that a narrow functional definition employing Granzyme B alone may, at least in some settings, not capture the entirety of responding CD4 T cells with cytotoxic potential. Indeed, Eomes^+^ Granzyme K^+^ cytolytic CD4 T cells have been reported in patients infected with Vaccinia virus [50], and a spectrum of cytolytic CD4 T cells, including an Eomes+ Granzyme K+ cohort as well as Eomes-T-bet+ cells, have been characterized in elite HIV controllers [51]. Further studies are required to delineate the relevance of this heterogeneity during immune responses in which cytotoxic CD4 T cells play important roles.

## 5. Eomes in Regulatory CD4 T Cells

Regulatory CD4 T cells (Tregs) are critical for preventing aberrant T cell responses and potentially damaging inflammation. Eomes deficiency in T cells has been shown to increase the number of Foxp3^+^ CD4 T cells that accumulate in aged mice [10]. The Eomes-deficient Foxp3^+^ CD4 T cells in this study appeared similar to WT Tregs in their expression of hallmark surface markers including CD25 and cytotoxic T-lymphocyte-associated antigen 4 (CTLA-4), and in their production of the immunosuppressive cytokine, IL-10. This suggests that Eomes does not directly impact the regulatory capacity of Tregs. Interestingly, the transfer of either naïve WT or Eomes-deficient CD4 T cells into immunodeficient mice in a model of colitis resulted many more Tregs developing in the mice receiving Eomes-deficient cells [10]. This suggests that Eomes may play an important role in preventing development of induced Foxp3^+^ Tregs during immune responses. Using Foxp3 reporter mice, Lupar et al. observed that ectopic expression of Eomes led to a reduced number of Foxp3^+^ CD4 T cells when stimulated in Treg-inducing culture conditions, further establishing a role for Eomes in restricting Treg differentiation in activated CD4 T cells [10].

Interestingly, Eomes may have an opposite role in regulating the development of Type 1 Regulatory (Tr1) cells, an important Foxp3^-^ CD4 T cell population marked by strong IL-10 production found in several disease states [52]. Eomes was shown to promote Tr1 development by promoting IL-10 production and preventing the development of other subsets in concert with B Blimp-1 in a mouse model of allogenic bone marrow transplantation [53]. Similar observations were made in a murine leukemia model, in which Eomes expression by CD4 T cells was shown to be critical for generating Tr1 cells that also exhibited cytotoxic function [54]. Together, with the results above focused on Foxp3^+^ Tregs, these results indicate that expression of Eomes by CD4 T cells may either positively or negatively impact suppressive modulation of ongoing immune responses depending on the predominating regulatory CD4 T cell subset.

## 6. Eomes and Th2 Cells

Th2 effectors characterized by the production of cytokines such as IL-4, IL-5, and IL-13 are important in orchestrating defenses against extracellular pathogens. The impact of Th2 regulators on Eomes expression in CD4 T cells appears to be complex. While IL-4, the critical driver of Th2 fate, can increase Eomes expression in effector CD8 T cells [22], Eomes is repressed by IL-4, and hence, is barely detectable in Th2-polarized CD4 T cells [9,55]. In line with this, CD4 T cells deficient for STAT6 (required for IL-4 signaling) and T-bet were found to upregulate Eomes mRNA expression more strongly than T-bet-deficient or WT cells [21]. This finding indicates that IL-4 signaling may restrict Eomes expression. Yet, CD4 T cells deficient for the transcription factor Krüppel-like Factor 2 (KLF2) show increased IL-4 production and high Eomes expression [56]. This suggests a circuit in which IL-4 mediated Eomes repression may require KLF2 signaling. The impact of Gata-3 expression on Eomes induction is also not entirely clear. On the one hand, CD4 T cells deficient for T-bet and STAT6 can co-express Gata-3 and Eomes [21]. However, WT cells retrovirally expressing Gata-3 and Runx3 grown under Th2 conditions fail to express Eomes, suggesting that Gata-3 may repress Eomes by preventing Runx3-mediated induction [24]. Taken together, these studies indicate that cooperativity between STAT6 and Gata-3 signaling pathways may be required to completely repress Eomes expression in Th2-polarizing environments.

Eomes has been implicated in the repression of Th2 development. For example, Gata-3-deficient CD4 T cells activated in Th2-polarizing conditions were found to produce IFNγ, which correlated with elevated levels of Eomes, but not of T-bet [24]. Eomes did not directly repress IL-4 production in these studies, but ectopic expression of Eomes did significantly reduce Gata-3 expression in WT cells grown under Th2 conditions [24]. Production of IL-5, a cytokine closely associated with regulating the maturation, activation, and proliferation of eosinophils, has also been shown to be negatively impacted by Eomes in CD4 T cells. Mechanistically, this was shown to result from Eomes-mediated capping of IL-5 production through preventing Gata3 from binding the *IL5* promoter [57,58]. This suggests that induction of Eomes in Th2 cells may offer therapeutic potential in settings such as allergic responses. 

## 7. Eomes and Th17 Cells

Th17 regulators appear to have a negative impact on Eomes expression in CD4 T cells. To understand regulatory mechanisms governing Th1 and Th17 development and to minimize interference from potential pro-Th2 IL-4 signals, Yang et al. studied CD4 T cells deficient for T-bet and STAT6, and observed that IFNγ production in these cells was dependent on Eomes [21]. However, exposure of the cells to IL-6 or Transforming Growth Factor-β (TGF-β), both critical inducers of Th17 development [59], repressed Eomes-dependent IFNγ production, which could be restored by ectopic Eomes expression [21]. Interestingly, while TGF-β signaling is most strongly associated with Smad transcription factors, TGF-β-dependent Eomes repression is Smad-independent and instead requires Jun-N-terminal kinases (JNKs) signaling [60,61]. Exposing developing Th1 CD4 T cells to IL-21, also linked with Th17 differentiation, was found to reduce their ability to produce IFNγ, with no impact on T-bet but strong reductions in Eomes protein and mRNA [9]. These observations support the assertion that Th17 regulators can suppress Eomes expression. 

T-bet has also been shown to repress Th17 development by interacting with Runx1, thus preventing the activation of the Th17 ‘master regulator’ RORγt [62]. Intlekofer et al. observed that lack of T-bet results in mixed Tc1/Tc17 responses by CD8 T cells in mice infected with lymphocytic choriomeningitis virus (LCMV), an intracellular murine pathogen. However, Tc17 responses were exacerbated, with near complete loss of IFNγ production, when T-bet and Eomes were deleted from T cells, indicating that both are repressors of Tc17 development [63]. We observed similar impacts of knocking out both T-bet and Eomes in unleashing strong protective Th17 responses against Influenza A Virus, as will be discussed further on.

T-bet and Eomes regulate genes by binding to specific T-box binding sites. Both *IL17A* and *RORC* have T-box binding sites in their promoter thus enabling Eomes to directly repress Th17 development [60,61,64]. Th17 polarization is directed by cytokines including IL-21, IL-23, IL-6 and TGF-β. The addition of these signals was found to reduce Eomes expression during in vitro Th17 development, while, on the other hand, ectopic Eomes expression was shown to abrogate Th17 development, further establishing a role for Eomes as a Th17 repressor [9,21,61]. It is important to note that the loss of Eomes alone in CD4 T cells does not result in ‘default’ increases in IL-17 production, as shown in studies utilizing Experimental Autoimmune Encephalomyelitis (EAE), the murine model of Multiple Sclerosis (MS) [10]. Thus, while inhibiting Eomes expression may be a critical first step for optimal Th17 development, it is not sufficient to promote Th17 polarization in the absence of other key inflammatory signals.

## 8. Eomes and Non-Classical Th1/Th17 Cells 

Immune responses including both Th1 and Th17 components are increasingly documented. Individual CD4 T cells responding in such environments can be marked by prototypical Th1 or Th17 attributes, but some cells also display a ‘non-classical’ mixed Th1/Th17 effector program. These non-classical cells may develop from Th17 cells in highly inflamed tissues. Eomes expression has been shown to support the development of effector cells expressing concurrent Th1 and Th17 functions by both promoting IFNγ production and restricting expression of IL-17 and Rorγt [64]. Indeed, in this study, WT Th17 cells primed in vitro were found to express more Th1 cytokines when transferred in vivo in a mouse model of colitis than did Th17 cells that could not express Eomes. Importantly, the enhanced Th1/Th17 phenotype driven by Eomes led to slightly more severe disease, suggesting that non-classical CD4 T cell effectors may have broader roles in driving damaging inflammation in the gut, and perhaps other organs.

Further studies will no doubt reveal additional signals outside of the scope of those tied to specific Th subset programs that impacts Eomes induction in CD4 T cells. One such factor is prolactin, which is best characterized as a stimulator of milk production released by the pituitary gland. However, prolactin was shown to strikingly induce Eomes in naïve CD4 T cells in vitro, with impacts of extrapituitary prolactin on Eomes expression in CD4 T cells demonstrated in vivo [65]. Another signal outside of standard Th subset-inducing signals that may induce Eomes in CD4 T cells is type I IFN. Indeed, type I IFNs strongly induce Eomes in naive CD8 T cells [66,67]. However, Eomes induction in CD4 versus CD8 T cells may be differentially impacted by the same signal, as seems to be the case for IL-12, as discussed above.

## 9. CD4 T Cell Memory Regulation by Eomes

Eomes appears to be important in coordinating the programming required for effector CD8 T cells to survive the contraction phase of immune responses and survive as long-lived memory cells [68]. CD8 T cells expressing higher levels of T-bet appear fated to become terminal effectors [69]. This suggests that the ratio of T-bet to Eomes is an important rheostat in controlling memory fate, with higher ratios promoting short-lived effectors and lower ratios promoting memory precursor effectors [70]. Similar observations of reduced memory potential from CD4 T cells expressing higher levels of T-bet have been reported [71]. An intriguing possibility, given data from studies of CD8 T cells, is that Eomes-dependent programming may be especially important in promoting memory formation from cells with lower affinities for antigen [72].

The relative expression level of T-bet by CD4 T cells responding to infection may play a particularly important role in establishing tissue-resident memory (T_RM_) populations, at least in the lung. Observations in mouse models of Influenza A virus (IAV) infection indicate a fitness advantage for CD4 T cells expressing lower levels of T-bet to compete for survival in the T_RM_ niche [29,73], a pattern also seen IAV-specific CD8 T cells [74]. Correlations between increased T-bet expression and decreased respiratory CD4 T_RM_ formation following viral infection have also been seen in clinical studies [75]. As Eomes is induced in lung CD4 T cells responding to IAV [18], these observations suggest that the T-bet/Eomes ratio may also be an important regulator of lung T_RM_ fate. Indeed, Eomes was found to be expressed at higher levels by CD4 T_RM_ than by CD4^+^ central and effector memory T cells primed by LCMV [76], and some CD4 T_RM_ in human lungs are marked by Eomes expression [77]. 

One of the mechanisms by which Eomes may promote CD4 T_RM_ is by regulating expression of the transcription factor Homologue of Blimp1 in T cells (Hobit), a control mechanism identified as necessary for supporting CD8 T_RM_ formation during LCMV infection [78]. Hobit has also been shown to direct intestinal CD4 T_RM_ formation, and increased expression of Eomes mark human CD4 T cells expressing Hobit [79,80]. These observations support the assertion that the Eomes–Hobit axis may be relevant more generally in CD4 T_RM_ generation. 

A second possible mechanism by which Eomes may improve T_RM_ fitness is by increasing the ability of CD4 T cells to compete for IL-15. The IL-2 receptor β chain (CD122), which is also a key component of the IL-15 receptor, is a direct target of Eomes [6]. We showed that lung CD4 T_RM_ primed by IAV can be supported by IL-15 signals [81], which is in contrast to virtually all circulating memory cells that require autocrine IL-2 signals to program memory fitness [82]. CD8 T cells and NK cells usually outcompete CD4 T cells for IL-15, which is largely trans-presented to them, by virtue of their higher expression of CD122 than CD4 T cells [83]. Thus, Eomes-driven upregulation of CD122 in CD4 T cell effectors may be an important event in promoting CD4 T_RM_ fitness through enhancing the ability of T_RM_ precursors to compete for a limiting survival signal. More experiments are required to test this possibility, and its potential to impact CD4 T_RM_ in other tissues.

## 10. Regulation of CD4 T Cell Exhaustion by Eomes?

Evidence from many lines of investigations support a role for Eomes in promoting CD8 T cell exhaustion [84,85,86,87]. Whether or not similar transcriptional regulation operates in CD4 T cells to promote an exhausted state is not as clearly understood. This possibility is supported by findings in colorectal patients in which a diverse array of molecular hallmarks of exhaustion were similarly expressed between CD4 and CD8 T cells expressing higher levels of Eomes [88]. Eomes expression is also upregulated in exhausted CD4 and CD8 T cells in the setting of chronic LCMV infection and chronic toxoplasmosis, [89,90]. These observations suggest that therapeutic upregulation of Eomes in disease-driving CD4 T cells may offer a strategy to alleviate chronic autoimmune conditions, such as Lupus. The validity of this approach is supported by recent studies indicating that higher levels of Eomes in CD4 T cells correlate with an exhausted phenotype in Lupus patients with long-standing remission versus CD4 T cells in patients with active disease [91].

CD4 T cells help during chronic infection and can prevent CD8 T cell exhaustion, and this outcome is correlated with preventing responding CD8 T cells from upregulating Eomes expression [90,92]. Thus, enhanced Eomes expression in CD4 T cells leading to an exhausted phenotype may also indirectly contribute to CD8 T cell exhaustion by preventing the delivery of optimal helper signals, ensuring robust function. On the other hand, stimulation with CD134 and CD137 agonists to boost Eomes expression in CD4 T cells was found to improve outcomes in a mouse model of tumor immunity; an outcome that correlated with increased anti-tumor CD8 T cell responses [93]. A more complete understanding of the signals and circuits regulating Eomes expression may thus provide broader strategies to modulate CD4 T cell responses to overcome dysfunctional cellular immunity in settings like chronic infectious disease and cancer.

## 11. Impacts of Eomes Expression by CD4 T Cells on Disease Outcomes

Aberrant CD4 T cell responses are associated with multiple immunopathologies, but they are also critical components of protective immune responses against cancers and pathogens [94,95]. Figure 2 broadly indicates the most relevant disease states that Eomes expression in CD4 T cells has been found to impact, linking them to functional ties with Eomes discussed earlier.

## 12. Eomes in Autoimmune Diseases

Several studies have shown a critical role of CD4 T cells in the pathogenesis of MS [95,96,97]. Multiple genotyping studies assessing potential Single Nucleotide Polymorphisms (SNPs) have established positive links between Eomes and MS [98,99,100,101]. Autopsies of MS patients also reveal higher frequencies of Eomes^+^ CD4 T cells with cytotoxic markers, a hallmark of the autoimmune disease, compared to healthy controls [101]. 

The Foxo3–Eomes axis has been shown to promote the development of pathogenic CD4 T cells in the mouse EAE model of MS. Mice deficient for Foxo3 show slower disease development than WT mice, which is linked to lower IFNγ production by the Foxo3-deficient CD4 T cells [11]. Eomes expression is positively regulated by Foxo3, as previously discussed, and lentiviral expression of Eomes in CD4 T cells has been shown to promote production of IFNγ and other cytokines associated with neuroinflammation in EAE, linking Eomes expression in CD4 T cells to pathogenicity [11]. Raveney et al. divided CD4 T cell-mediated EAE into two phases: an early phase dependent on Th17-linked nuclear receptor 4A2 (NR4A2) and a later phase dependent on Eomes-mediated Granzyme B expression in CD4 T cells [102]. The severity of the late phase was significantly reduced in mice deficient in both NR4A2 and Eomes, and was linked to lower Granzyme B expression by Eomes-deficient CD4 T cells. Additionally, Eomes levels have been found to be higher in CD4 T cells present in the cerebrospinal fluid versus in the blood of MS patients [102]. Likewise, analysis of synovial fluid from patients with Juvenile Idiopathic Arthritis also revealed more Eomes^+^ CD4 T cells than present in the blood [64]. These observations indicate that Eomes-dependent expression of adhesion molecules and chemokine receptors may contribute to site-specific accumulation of effector CD4 T cells at sites of inflammation. The receipt of IL-15 signals by CD4 T cells responding in MS patients may be important in this process. As discussed above, Eomes activation can upregulate surface expression of CD122 thereby making CD4 T cells more competitive to access IL-15. IL-15 is produced in inflammatory MS lesions, can induce expression of specific adhesion molecules and chemokine receptors on CD4 T cells derived from patient blood, and induce in them the expression of cytotoxic molecules, including perforin and Granzyme B [103].

Another mechanism by which Eomes may promote immunopathology is by inhibiting the development of Foxp3^+^ Tregs. Indeed, Eomes-deficient bystander cells co-transferred with encephalitogenic TcR transgenic CD4+ T cells were shown to develop more efficiently into Foxp3^+^ Tregs that could suppress EAE than could WT bystander cells [10]. Further studies are required to test if increased Eomes expression by responding CD4 T cells promotes increased immunopathology in other settings by restricting induced Treg differentiation. 

Colitis is a chronic inflammatory disease linked to inflammation-triggered responses of Th1 and Th17 cells [64,104]. A functional shift from a Th17-dominated to a Th1-dominated response appears to be critical for severe disease in at least some colitis models. Tamoxifen-induced Eomes deletion in Th17-polarized cells introduced into immune-deficient mice reduced the total influx of cells into the colon, while Th17 cells overexpressing Eomes were found to display Th1 and cytotoxic attributes associated with disease progression [64]. Further studies are required to test if a role for Eomes in promoting a pathogenic Th17 to Th1/cytotoxic functional shift may similarly impact other inflammatory disorders.

In contrast to the studies summarized above, Eomes may have a positive role in repressing airway allergic responses mediated by IL-5-producing Th2 cells. Eomes has been shown to negatively regulate memory Th2 cells that produce IL-5. Indeed, memory Th2 CD4 T cells generated in vitro and deficient in Eomes showed enhanced IL-5 production and significant increased inflammatory cell infiltration as compared to WT cells in a murine model of airway inflammation. These observations demonstrate that by preventing airway hyperinflammation, Eomes induction can have a protective role in CD4 T cell-driven allergic responses [57]. 

## 13. Eomes in Cancer Immunity

Several studies correlate Eomes expression with improved protection against cancers by CD4 T cells [16,42,105,106,107,108]. Most often, increased cytolytic capacity has been linked to the protective impacts of CD4 T cells expressing higher levels of Eomes [15,16,109]. As discussed earlier, therapeutic CD137 and CD134 co-stimulation can promote Eomes expression, and this treatment has been shown to program a cytotoxic phenotype able to eliminate cancer cells in mice [14,15,16]. CD137 co-stimulation has also been shown to reprogram Tregs into effective Eomes-expressing cytolytic cells [110]. Notably, CD4 T cells with cytolytic signatures and high Eomes expression are found in patients with neuroblastoma, a cancer where CD4 T cells are often more effective than CD8 T cells at promoting tumor destruction [111]. Eomes has also been implicated in programming other CD4 T cell subsets, such as Th9 cells, to become cytolytic and eliminate tumor cells [112]. That high Eomes expression is increasingly observed in T cells at tumor sites and is positively correlated with increased survival in patients, suggests that its actions may go beyond promoting cytolytic activity in promoting CD4 T cell-mediated protection [109,113,114].

While T cells are promising antitumor mediators, their dysfunction is also hallmark in cancerous tissues. Several studies have shown that tumor cells can escape immune surveillance by engaging inhibitory molecules on T cells such as CTLA-4, programmed death-1 (PD-1), and CD39 [115,116]. A strong correlation between lower Eomes expression in T cells at tumor sites and poor prognosis was seen in patients with Hepatocellular carcinoma [117], suggesting that high expression of inhibitory molecules may negatively impact Eomes-dependent functions. In support of this hypothesis, cancer patients given anti-CTLA-4 and anti-PD-1 therapy develop tumor-specific cytolytic CD4 T cells marked by enhanced Eomes expression [118]. Eomes may promote protective CD4 T cell activity by directly binding the regulatory regions of inhibitory molecules, thus lowering their expression on effector cells and increasing their effectiveness at tumor sites [117]. Conversely, Eomes^+^ Tr1 cells expressing cytolytic markers and PD-1 have been found in patients with chronic lymphocytic leukemia (CLL) [54]. While the role of these cells in CLL patients is unclear, studies using mouse models show that Tr1 cells can control tumor growth through IL-10 production and Eomes-mediated cytolytic activity [54]. 

It is important to note, however, that CD4 T cells marked by high levels of Eomes do not always correlate with improved outcomes. For example, Eomes expression by CD4 T cells was recently found to mark responses of colorectal cancer patients with shorter overall survival [88]. Cells with higher levels of Eomes expressed many cellular markers of functional exhaustion as well as several immune checkpoint inhibitory molecules. As Eomes is associated with both exhausted CD4 T cells and with effectors with improved anti-tumor function, interpretation of its expression in terms of clinical prognosis can be challenging. Thus, a deeper understanding of its regulation in CD4 T cell effectors at tumor sites may lead to improved immunotherapies and prognostic tools.

### 13.1. Eomes in Antiviral Responses

Eomes has been shown to impact CD4 T cell responses against diverse viral infections. For example, CD4 T cell responses against Human Immunodeficiency Virus (HIV), Cytomegalovirus (CMV) and Epstein–Barr Virus (EBV), characterized by cytotoxic functions are important contributors to host defenses [17,119,120]. HIV predominantly affects and depletes CD4 T cells, leading to a gradual loss of immune fitness and susceptibility to opportunistic pathogens and cancers [121]. CD4 T cells with a cytolytic profile have been correlated with improved viral control in HIV patients [122]. Buggert et al. identified CD4 T cells with a protective profile as T-bet^High^ and Eomes^+^ [123]. Similarly, Johnson et al. observed that Eomes^+^ cytolytic CD4 T cells emerged during early viremia and that these effectors were comparable to CD8 T cells in their ability to control the virus [124]. Interestingly, Nicoli et al. found that the HIV protein, Trans-Activator of Transcription, can stimulate the development of Eomes^high^ expressing CD4 T cells [125].

Protective CD4 T cell responses in patients with primary EBV infections, a potential tumorigenic virus, have also been correlated with a cytotoxic phenotype marked by high expression of Granzyme B, perforin, and Eomes [119]. Using a mouse model of EBV infection, Choi et al. show that the EBV surface protein, latent membrane protein 1 (LMP1), mediated stimulation of the co-stimulatory molecules CD137 and CD134 that, as discussed previously, can promote strong Eomes induction in CD4 T cells [17]. CMV is an opportunistic pathogen that causes life-threatening disease in donor organ recipients and immunocompromised individuals [126]. Latent CMV replication is kept under control by continuous T cell immunosurveillance [127]. Pera et al. characterized effective CD4 T cell responses against CMV as cytolytic, with strong Eomes expression [128]. Additionally, Eomes expression in older CMV-seropositive individuals may be critical to prevent progression into an active infection [129]. These observations suggest that targeting Eomes induction in CD4 T cells may potentially serve as strategy to promote immunity against many different viral pathogens. 

### 13.2. Eomes in CD4 T Cell Responses against Influenza A Virus

**Figure 3:**
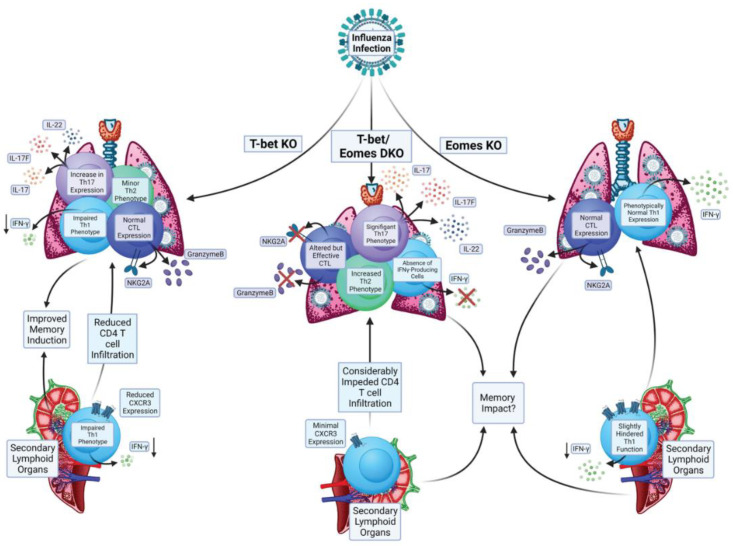
Impacts of Eomes and its paralog T-bet on CD4 T cell responses during Influenza A Virus infection.

We recently investigated the impact of Eomes expression by CD4 T cells responding to IAV because the infection drives effector responses marked by cytokine production and cytotoxic activity [130] that have been shown to be impacted by Eomes in other models, as discussed above. We were also interested in studying Eomes because we had previously characterized the impact of T-bet-deficiency in CD4 T cells during anti-IAV responses. In this work, we found that, compared to WT anti-viral CD4 T cell responses, T-bet^-/-^ cells showed impaired, but still relatively robust, Th1 attributes (Figure 3). Most notably reduced in T-bet^-/-^ CD4 T cells were IFNγ production, expression of the chemokine receptor CXCR3, and expression of the activation marker Lymphocyte antigen 6 complex (Ly6C) [29]. The T-bet^-/-^ cells also developed a cohort of Th17 cells (Rorγt^+^IL-17^+^) and a smaller cohort of Th2 cells (Gata3^+^IL-4^+^) that were not present in responding WT CD4 T cells. Perhaps surprisingly, production of IL-10, which is largely restricted to IFNγ^+^ CD4 T cells in the lungs of IAV-infected mice [131], was not impacted by T-bet expression, nor were cytolytic CD4 T cells marked by Granzyme B and Natural Killer group 2 member (NKG2) A/C/E expression [29]. Several of these hallmarks were also seen in a separate study comparing the T cell responses of WT and T-bet^-/-^ mice after IAV infection [132].

In comparison to the patterns above, we found that the impact of knocking out Eomes in CD4 T cells responding to IAV was much more limited (Figure 3). The Th1-dominated cytokine response seen from WT cells in the lung was largely intact in Eomes^-/-^ cells, as was the production of IL-10 and cytotoxic function [18]. These results are consistent with other observations in the mouse model of IAV indicating that Eomes is not required for Granzyme B expression in CD4 T cells [32], and finding no differences in Eomes expression between cytolytic and non-cytolytic CD4 T cell lung effectors [133].

However, IFNγ production was significantly, albeit slightly, reduced in Eomes^-/-^ CD4 T cells responding in the secondary lymphoid organs of IAV infected mice. We measured Eomes levels in the IAV-primed WT cells, and while it was induced in effectors responding in the lung, Eomes was higher in effectors present in the spleen and draining lymph nodes [18]. These results indicate organ-specific expression patterns of Eomes by CD4 T cells, with corresponding impacts on cytokine production capacity. This regulation may be important to consider more generally when assessing the potential for Eomes to affect CD4 T cell responses in other mouse models and in biological samples from patients. Further studies are needed to identify the cell extrinsic and intrinsic factors that impact Eomes induction in the cells responding at different sites during infection.

Based on these results, we hypothesized that expression of either T-bet or Eomes may be sufficient to program key anti-viral attributes (generally associated with Th1 programming). To test this, we bred T-bet^-/-^Eomes^-/-^ double knockout (DKO) mice. We found that IAV-specific DKO CD4 T cells lost virtually all capacity for IFNγ production and displayed a dramatic reduction in the number of cells expressing molecules associated with cytotoxicity [18]. These results indicate that the residual Th1 attributes of T-bet^-/-^ CD4 T cells responding against IAV are largely Eomes-dependent. However, as compared to regulation of hallmark cytolytic CD8 T cell functions for which T-bet and Eomes are largely redundant, our results highlight that in the absence of T-bet, Eomes-dependent programming can only partially support Th1 differentiation in response to viral infection.

Surprisingly, we found that DKO CD4 T cells retained the capacity to protect naive mice against lethal IAV infection despite losing their Th1 imprint [18]. Our analysis revealed that the DKO cells developed an extremely strong Th17 phenotype, supporting the hypothesis that Th17 responses can protect against IAV, even in the absence of Th1 response elements. Interestingly, while our results showed that DKO mice can clear primary and heterosubtypic IAV challenges with similar kinetics of viral clearance to WT mice, with no overt signs of enhanced immunopathology, earlier studies found that DKO mice rapidly succumb to LCMV challenge. This lethal outcome was driven by strong IL-17 production, particularly by CD8 T cells responding to LCMV [63]. Combined, these results indicate that unadulterated Th17 responses, while detrimental and potentially lethal in the setting of some viral infections (like LCMV), can also be highly protective against other viruses (like IAV).

## 14. Summary

Eomes is emerging as a transcription factor that can modulate several aspects of effector responses mediated by CD4 T cells. As Eomes also appears to impact memory T cell fitness, its differential expression may be an important contributing factor to the heterogeneity observed within activated CD4 T cell populations in vivo [134]. This suggests that modulating Eomes expression could improve vaccination or immunotherapy regimes incorporating CD4 T cells and serve as a target to alleviate CD4 T cell-driven autoimmune conditions like MS. However, a complication to such approaches is that the impact of Eomes on CD4 T cells appears to vary dramatically, depending on the experimental model or disease state under investigation. Future studies will no doubt reveal further aspects of CD4 T cell immunobiology that can be impacted by Eomes and provide more insight into how its various impacts are regulated For example, an intriguing possibility, suggested by studies with CD8 T cells, is that Eomes may impact chromatin remodeling in CD4 T cells, and thus, contribute to epigenetic regulation impacting effector functions and memory fate [135,136]. A more comprehensive understanding of the full scope of signals that modulate Eomes expression and that are impacted by its expression represents an important goal that could provide novel strategies to optimize CD4 T cell immunity.
